# The Interest in Permanent Contraception Peaked Following the Leaked Supreme Court Majority Opinion of Roe vs. Wade: A Cross-Sectional Google Trends Analysis

**DOI:** 10.7759/cureus.30582

**Published:** 2022-10-22

**Authors:** Armin Ghomeshi, Parris Diaz, Valencia Henry, Ranjith Ramasamy, Thomas A Masterson

**Affiliations:** 1 Urology, Florida International University, Herbert Wertheim College of Medicine, Miami, USA; 2 Urology, University of Miami, Miami, USA; 3 Urology, Edward Via College of Osteopathic Medicine, Spartanburg, USA

**Keywords:** vasectomy, tubal ligation, roe v. wade, google trends, contraception, abortion

## Abstract

Introduction

With the leaked Supreme Court draft regarding Roe vs. Wade, substantial public reactions followed as the horizon of abortion laws within the United States of America have been changing. We sought to compare internet searches for vasectomy and tubal ligation seven days following the leaked draft on May 2, 2022.

Methods

We used public data provided by Google Trends to investigate the interest and geographic distribution of searches for the two forms of permanent contraception: Vasectomy and tubal ligation. We calculated the mean Search Volume Index (SVI) of these terms. Data analysis was performed with Microsoft Excel Version 16.60 (Redmond, USA), and comparisons between groups were performed using paired t-tests.

Results

The term 'vasectomy' saw a 121% increase (p=0.0063), and 'tubal ligation' had a 70% (p=0.029) increase compared to the week prior. 49/50 states had increased search inquiries for each term. However, the North and Southwestern regions of the U.S. had increased relative surge for vasectomy and the Midwest region for tubal ligation procedures, respectively. South Dakota and Idaho, with trigger laws that banned abortion immediately following the overturn of Roe vs. Wade, had the greatest surge in SVI for tubal ligation and vasectomy, respectively.

Conclusion

Our study indicates that with the potential overturn of Roe vs. Wade, there was a significantly increased interest in these two forms of permanent contraception. Future studies should investigate specific concerns and questions patients may have when it comes to the different options of contraception.

## Introduction

Roe vs. Wade, 410 U.S. 113 (1973) constitutionally protected a woman’s right to privacy regarding reproductive health, autonomy over one’s own body, and the right to abortion without government interference. Associate Justice Samuel Alito's draft opinion, leaked from the U.S. Supreme Court on May 2, 2022, opens with these words: "Abortion presents a profound moral issue on which Americans hold sharply opposing views" [1]. Despite the existence of this constitutional right since 1973, state and local governments have attempted to abolish it and limit its rights by enforcing bans based on the duration of pregnancy [2]. In addition, the Hyde Amendment blocked Medicaid from funding abortions except for extenuating circumstances such as rape or incest [3]. While abortion has been a subject of contention between political parties for years, the laws protecting the right to abortion have generally been upheld. However, quotes from the leaked draft from the Supreme Court, such as "Roe was egregiously wrong from the start" and "We hold that Roe and Casey must be overruled," hinted at the overturning of this constitutional right [4]. This decision will affect the availability of safe medical abortions for thousands of women across the country since each state has the authority to decide when and whether abortion is legal. Currently, 13 states (Arkansas, Idaho, Kentucky, Louisiana, Mississippi, Missouri, North Dakota, Oklahoma, South Dakota, Tennessee, Texas, Utah, and Wyoming) have triggered laws that made abortion illegal as soon as Roe was overturned [5]. Previous literature suggests that the legality of abortion influences contraception usage and the interest in possible sterilization. As of 2018, the sterilization rate for women in the United States was about 18%, and as of 2009, the global sterilization rate for women was about 19%. However, this rate was significantly higher in countries with strict abortion laws, such as El Salvador (70%) and Dominican Republic (40%) [6-8]. The estimated rate of men aged 18-45 undergoing vasectomies in the United States between 2015-2017 was 4% [9]. With the criminalization of abortion in the United States, there may be a greater focus on preventing pregnancy among both women and men. Subsequently, this may result in a decline in birth rates, premature sterilization as well as an increase in maternal mortality [10-11].

Currently, there are various forms of contraception and sterilization in the United States, including birth control such as barrier protection with condoms, oral contraception pills, subdermal implants, intrauterine device (IUD), vasectomy, and tubal ligation surgery [12-14]. A vasectomy is a procedure where the tube that carries sperm from each testicle (vas deferens) is cut and sealed, and a tubal ligation involves cutting, tying, or blocking the fallopian tubes to prevent pregnancy [15,16]. With the Supreme Court’s overturn of Roe vs. Wade, contraception may be a more significant concern to people across the country. We hypothesized that there would be a greater interest in primary forms of sterilization, such as vasectomies and tubal ligation, following the leaked decision. To explore this, we sought to compare internet searches for vasectomy and tubal ligation seven days following the leaked draft on May 2, 2022. With the utilization of Google Trends, we sought to objectively measure trends in internet search queries on permanent contraception - specifically vasectomies and tubal ligations.

## Materials and methods

A cross-sectional retrospective study was conducted to investigate the influence of the leaked draft indicating the overturning of the Roe vs. Wade decision by the Supreme Court on online queries regarding sterilization procedures for men and women. A waiver was obtained from the Institutional Review Board (IRB), as Google Trends is considered publicly available data and qualifies for Institutional Review Board (IRB) exemption. Google Trends is a website created in 2006 that provides an analysis of online search queries made by users on the Google search engine over a given period (https://trends.google.com/trends) [17].

Search queries

On May 18, 2022, we investigated the search volume of the keywords ‘vasectomy’ and ‘tubal ligation’ across the United States between April 25, 2022 and May 8, 2022. These terms were chosen based on the popularity of these forms of permanent contraception by men and women, respectively. The popularity of each search term was explored one week before (April 25, 2022, to May 1, 2022) and after (May 2, 2022, to May 8, 2022) the leakage of the Supreme Court document on May 2, 2022. The interest in a keyword utilizes population-adjusted data to reflect search popularity at a given point in time [18]. Search Volume Index (SVI), provided by Google Trends, is a measure of interest in a keyword, calculated as the proportion of searches in each location and time divided by all searches [19-20]. The SVI of each keyword can range from a minimum of 0 to a maximum of 100, where 100 is defined as the peak search interest at that time and location [19]. During our investigation using Google Trends, each query was performed using the ‘search term’ capability to generate specific information on that keyword and exclude synonyms or translations of our keyword [21]. Geographic information for our established search terms ‘vasectomy’ and ‘tubal ligation’ were also queried to identify US regions of highest SVI between April 25, 2022 to May 8, 2022. Google Trends does not provide information on an individual's age, race, and ethnicity. 

Outcomes

The SVI of both keywords was evaluated between April 25, 2022, and May 8, 2022. The primary outcome was the difference in mean SVI one week before and after the Supreme Court document that was released on May 2, 2022. In addition, we aimed to describe the geographic areas in the United States with increased interest in our keywords. 

Statistical analysis plan

Data analysis was performed with Microsoft Excel Version 16.60 (Redmond, USA). Comparison of the SVI of 'vasectomy' and 'tubal ligation' one week before and one week after were performed with paired t-tests. A p < 0.05 was considered statistically significant.

## Results

**Figure 1:**
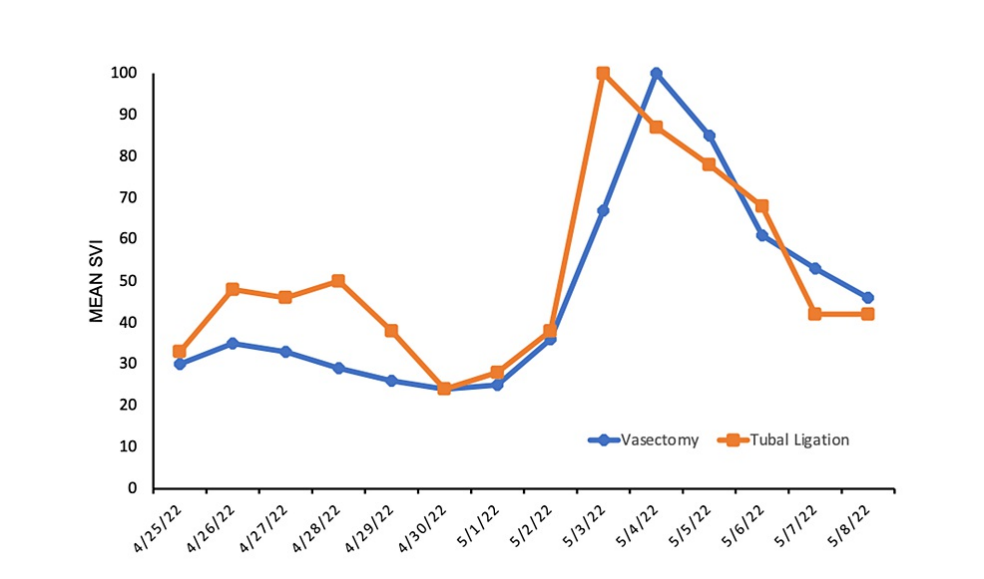
Mean Search Volume Index (SVI) of keywords 'Vasectomy' and 'Tubal Ligation' between April 25th - May 8th, 2022.

Two sterilization procedures, ‘Vasectomy’ and ‘Tubal Ligation’, were selected as keywords and subsequently analyzed. Google Trends revealed that both keywords saw a significant increase in their respective search volume in the seven days following the leaked decision suggesting the overturning of Roe vs. Wade on May 2, 2022. The mean SVI of the keyword ‘vasectomy’ was previously 28.9 but increased to 64 (+121%) (p=0.0063). ‘Tubal ligation’, a female sterilization procedure, saw a 70% increase from an SVI of 38.1 to 65 (p=0.029) (Figure 1).

**Figure 2:**
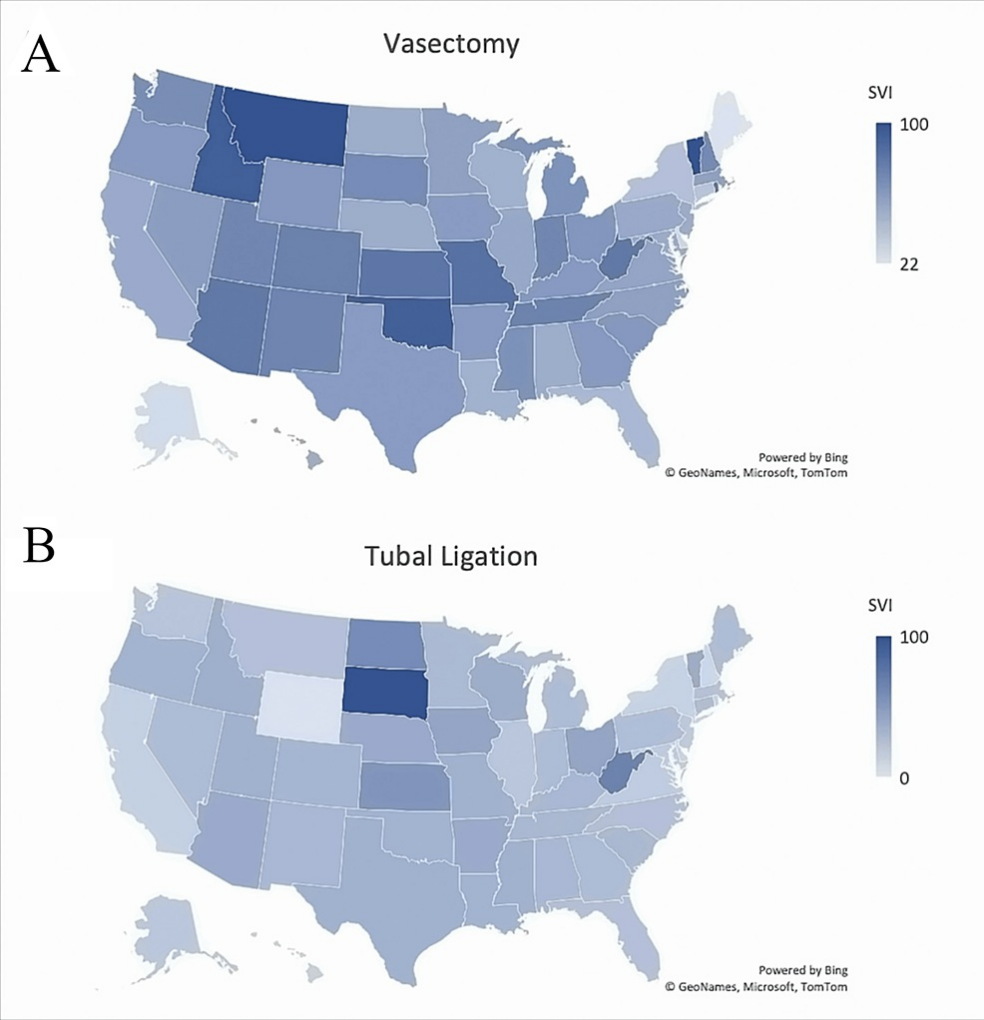
Geographical maps demonstrating Mean Search Volume Index (SVI) using the keyword and location (A) 'Vasectomy' in the U.S. and (B) 'Tubal Ligation' in the U.S.

When observing national trends, most U.S. states saw an increase in search volume during this period. Interestingly, those searching for the keyword ‘vasectomy’ were more often in the North and Southwestern regions, while tubal ligation was most often searched in the Mid-West (Figures 2A, 2C). South Dakota and Idaho, two trigger states, had a relative increase in SVI for tubal ligation and vasectomy, respectively (Figures 2A, 2C).

## Discussion

Using Google, the most popular internet search engine, we decided to identify potential changes in the interest of permanent contraception following the leaked decision to possibly Roe vs. Wade. Specifically, we wanted to gauge interest in vasectomies and tubal ligations seven days before the leak and seven days after to see to determine any changes in the interest of sterilization. The results of this study indicated that there was a statistically significant increase in internet search queries following the leak on May 2, 2022. Further analysis revealed two trigger states (South Dakota and Idaho) with a relative surge in these terms, possibly suggesting that inhabitants in these regions may have greater concern and interest in alternate forms of permanent contraception given the more immediate legal implications of the Roe vs. Wade ruling. 

Ultimately, people across the country are now faced with new challenges when it comes to pregnancy and family planning. The results of our study highlight the importance of disseminating accurate medical advice, as the internet is not always the best source of information, as suggested by Wu et al. [22]. With this data, physicians may now be aware that people in their communities are curious about new forms of contraception, including permanent methods. Previous literature has shown that up to 50% of women between 18-50 years of age were interested in discussing contraception with their primary care providers (PCPs) [23]. Interestingly, it was also reported that women “in need of counseling” were less likely to receive the proper guidance [23]. Our results indicate that the number of individuals making appointments for contraception counseling may potentially increase, thus creating an urgent need for fertility-related patient care. Physicians can work on tailoring their patient visits to address the individual needs of those who are interested in permanent methods of contraception. As the climate regarding abortion continues to evolve, physicians should be prepared to provide accurate medical counseling, answer specific questions, and dispel potential misconceptions patients may have obtained through the internet. To supplement these individual visits, public health measures, such as public seminars, can be put in place to further educate people on a macroscopic scale-especially for those who may not have regular access to a physician for counseling whether it be due to geographical or financial constraints. In summary, these plans of action can allow patients to make well-informed decisions when planning for pregnancy.

This study is not without strengths and limitations. Strengths of our study include the analysis of all regions in the United States where political and religious views on abortion can significantly differ. We also included two forms of permanent contraception that are performed in males and females to control for sex bias. On the other hand, online search inquiries may not completely reflect an individual’s true intent in proceeding with a vasectomy or a tubal ligation. Furthermore, Google Trends is unable to provide any specific demographics information of users, such as age, race, and ethnicity. The lack of this data prohibits us from identifying who in the community is most interested in learning or concerned about these procedures. Additionally, our study is limited to those who use Google as their search engine, and excludes those who prefer other search engines such as Yahoo, Bing, etc. [19]. This study also examined queries over a 14-day time frame, but more long-term data is needed to develop a more comprehensive conclusion about patient concerns with abortion now illegal in some regions of the United States. Finally, we included only two permanent methods of contraception and did not consider those who may be interested in alternative forms of birth control, including those that are irreversible.

## Conclusions

In summary, our study is one of the first to identify a surge in search-related inquiries in different forms of permanent contraception, specifically tubal ligation and vasectomy. These results allow physicians and public health officials to have a holistic insight into what patients are most interested in regarding the ongoing Roe vs. Wade situation. Future research should be done to examine how this decision will impact reproductive and sexual health for both females and males in the years to come. Given the increased interest in these procedures, it may fall upon the shoulders of non-governmental institutions to teach young men and women safe sex measures to prevent unplanned pregnancies. Healthcare providers should continue to educate themselves on the existing contraceptive and abortive methods to provide equitable counseling and access for their patients regardless of the choices they make. Finally, with Roe vs. Wade officially overturned, there may be a need to increase the number of physicians trained to perform vasectomies and tubal ligations, should the demand for these procedures increase.
